# Molecular epidemiology of cryptococcal genotype VNIc/ST5 in Siriraj Hospital, Thailand

**DOI:** 10.1371/journal.pone.0173744

**Published:** 2017-03-21

**Authors:** Chanin Hatthakaroon, Sujiraphong Pharkjaksu, Piriyaporn Chongtrakool, Kamol Suwannakarn, Pattarachai Kiratisin, Popchai Ngamskulrungroj

**Affiliations:** Department of Microbiology, Faculty of Medicine Siriraj Hospital, Mahidol University, Bangkok Noi, Bangkok, Thailand; Defense Threat Reduction Agency, UNITED STATES

## Abstract

Despite the strong association between *Cryptococcus neoformans* infection and the *Human immunodeficiency virus* (HIV) status of patients globally, most cryptococcosis cases in Far East Asia occur in non-HIV individuals. Molecular epidemiological studies, using multilocus sequence typing (MLST), have shown that more than 95% of cryptococcal strains belong to a specific subtype of VNI. However, this association has never been specifically examined in other parts of Asia. Therefore, in this study, we investigated the VNIc/ST5 genotype distribution among cryptococcosis patients in Thailand. Fifty-one *C*. *neoformans* isolates were collected from clinical samples in Siriraj Hospital, Bangkok, Thailand. The strains were predominantly isolated from HIV-positive patients (88.57%) and all were molecular type VNI MATα. An MLST analysis identified five sequence types (ST) in Siriraj Hospital, of which ST4 (45.10%) and ST6 (35.29%) were most common, and ST5 (15.69%), ST32 (1.96%), and ST93 (1.96) were less common. Contrary to reports from Far East Asia, ST5 was predominantly (83.33%) found in HIV patients (P = 0.657), and there was no significant change in the prevalence of ST5 over the past 10 years (P = 0.548). A further analysis of comorbidities showed higher morbidity and delays in the cryptococcal diagnosis in patients with tuberculosis coinfection or without HIV. Our study suggests that although the Thai population is genetically closely related to the Far East Asian population, ST5 is not associated with non-HIV status in Thailand. Therefore, this association may not be related to the host’s genetic background. However, its mechanism remains unclear.

## Introduction

Members of the *Cryptococcus* species complex include two major species, *C*. *neoformans* and *C*. *gattii*. *Cryptococcus* is an important opportunistic human pathogen causing life-threatening meningitis, with significant morbidity and mortality. *C*. *neoformans* and *C*. *gattii* are the major causative agents of human and animal cryptococcosis. *C*. *neoformans* is known to infect mainly immunocompromised hosts, whereas immunocompetent hosts are usually infected by *C*. *gattii* [1, 2]. *Cryptococcus* infection occurs after the inhalation of infectious propagules (basidiospores or blastoconidia), which primarily colonize the lung and subsequently invade the central nervous system (CNS) [3]. The yeast can survive in humans by expressing virulence factors such as the capsule protein, melanin, its growth at 37°C, and has a unique mating system.

PCR fingerprint, Restriction fragment length polymorphism (RFLP) analysis of the orotidine monophosphate pyrophosphorylase (*URA5*), Multilocus microsatellite typing (MLMT) and Multi-Locus Sequence Typing (MLST) analysis have been used to classify *C*. *neoformans* and *C*. *gattii* into eight major molecular types: VNI (var. *grubii*, serotype A), VNII (var. *grubii*, serotype A), VNIII (serotype AD), VNIV (var. *neoformans*, serotype D), VGI, VGII, VGIII, and VGIV (*C*. *gattii*, serotypes B and C). VNI is the most common molecular type among the strains collected from cases of clinical infection and is the leading cause of mortality among HIV patients worldwide, including in Thailand [4, 5]. The *Cryptococcus neoformans* was believed to cause opportunistic infections but cryptococcosis in immunocompetent patients has been increasingly reported. The ongoing outbreaks of cryptococcosis on Vancouver Island, Canada, and in the Pacific Northwest area of the USA have seen *C*. *gattii* emerge as a primary pathogen [6].

Furthermore, although the VNI-type *C*. *neoformans* was previously believed to predominantly infect immunocompromised patients. It is also reported to cause disease in healthy people [7]. Cryptococcosis caused by *C*. *neoformans* in immunocompetent hosts has been increasingly reported in Far East Asia regions, including China and South Korea, where 77.4%–100% of cryptococcosis infections occur in non-HIV patients. Multilocus sequence typing (MLST) has shown that more than 90% of these cases are infected by a specific subtype of molecular type VNI, the ST5 genotype. Although the overall association between ST5 infection and non-HIV status was reported again in a recent Asian study [8], this association has never been evaluated in other parts of Asia. Therefore, in this study, we investigated the association between HIV status and ST5 infection in Thailand, a Southeast Asian country.

## Materials and methods

### Clinical isolates

Cryptococcal isolates from 2012–2014 were obtained from clinical samples in the Department of Microbiology, Faculty of Medicine Siriraj Hospital, Mahidol University, Bangkok, Thailand. All the isolates were identified with the RapID^™^ Yeast Plus System (Thermo Fisher Scientific, MA, USA). L-Canavanine–glycine–bromothymol blue (CGB) agar was used to differentiate *C*. *neoformans* from *C*. *gattii*. Clinical isolates were collected upon approval from Siriraj Institutional Ethics Committee (No.SI 405/2014).

### Reference strains

A set of standard laboratory reference strains representing each of the eight major molecular types were used for molecular typing: WM148 (VNI), WM626 (VNII), WM 628 (VNIII), WM 629 (VNIV), WM 179 (VGI), WM 178 (VGII), WM 175 (VGIII), and WM 779 (VGIV) [3]. KN99α and KN99a were used as the reference strains for the mating-type analysis [9]. The reference strains were obtained through the courtesy of Dr. Wieland Meyer, The University of Sydney of Westmead Hospital, Westmead, NSW, Australia.

### Genotype and mating-type analyses

DNA was extracted with the phenol–chloroform–isoamyl alcohol (25:24:1, v:v:v) method [10]. The *URA5* gene was amplified with the following primers URA5 (5’ATGTCCTCCCAAGCCCTCGACTCCG3’) and SJ01 (5’TTAAGACC TCTGAACACCGTACTC3’). Genotypes were determined with a restriction fragment length polymorphism (RFLP) analysis of the *URA5* gene with the enzymes *HhaI* and *Sau96I* (Thermo Fisher Scientific, MA USA) [3]. Mating types were identified with PCR using two specific primers, STE20Aα and STE20Aa, as described previously [11].

### MLST

An MLST analysis of the *C*. *neoformans* isolates was performed, using the International Society for Human and Animal Mycology (ISHAM) consensus scheme of seven unlinked loci (*CAP59*, *GPD1*, IGS1, *LAC1*, *PLB1*, *SOD1*, and *URA5*). Each locus was amplified with previously described primers and amplification parameters [3, 4]. The allele types and sequence types (STs) were defined according to the ISHAM MLST database for *C*. *neoformans* (http://mlst.mycologylab.org) [2]. The generated sequences were manually edited using the software and aligned using Clustal W. The concatenated alignments were then imported to the program MEGA 6.06 and analyzed using the neighbor-joining method with p-distance. Bootstrap analysis using 1000 replicates was used to estimate support for clades of the concatenate dataset.

### Statistical analysis

The statistical analysis was performed with Fisher’s exact test using the online program SISA (http://www.quantitativeskills.com/sisa/statistics/fisher.htm). Unknown data were regarded as lost and were not included in the calculations. Statistical significance was defined as P-value ≤ 0.05.

## Results

### Distribution of major molecular types, mating types and demographic data

A total of 165 *C*. *neoformans* isolates were collected from 51 patients. Only one isolate per patient was selected for analysis because, typically, serial isolates from the same patient belong to the same genotype [12]. Moreover, a recent study from Thailand also revealed all serial isolates belonged to the same genotype [4]. Cerebrospinal fluid (CSF) was the most common site of isolation, followed by blood and bone marrow (Table 1). This result was in line with previous studies that *C*. *neoformans* was majorly associated with meningitis [4]. All the isolates were identified as molecular type VNI and mating type MATα (Table 2).

**Table 1:** Characteristics of *C*. *neoformans* isolated from patients.

Characteristics	Count (%)
**Isolation sites (n = 51)**	
• Cerebrospinal fluid	29 (56.86%)
• Blood	21 (41.18%)
• Bone marrow	1 (1.96%)
**Patient’s ages (n = 35)**	
• 0–15 years	0
• 15–60 years	29 (82.86%)
• >60 years	6 (17.14%)
**Gender (n = 35)**	
• Male	23 (65.71%)
• Female	12 (34.29%)
**HIV status (n = 35)**	
• Positive	31 (88.57%)
• Negative	4 (11.43%)

**Table 2:** Alleles and sequence types of the cryptococcal isolates in this study.

Strain	Isolation site	HIV status	Molecular type	Mating type	Allelic profiles							ST
					*CAP59*	*GPD1*	IGS1	*LAC1*	*PLB1*	*SOD1*	*URA5*	
SICN_001	CSF	+	VNI	α	1	3	1	5	2	1	1	5
SICN_002	Blood	-	VNI	α	1	1	1	4	2	1	5	4
SICN_003	Blood	+	VNI	α	1	1	1	3	2	1	5	6
SICN_004	CSF	+	VNI	α	1	1	1	4	2	1	5	4
SICN_005	Blood	+	VNI	α	1	1	1	4	2	1	5	4
SICN_006	Blood	+	VNI	α	1	1	1	4	2	1	5	4
SICN_008	Blood	-	VNI	α	1	1	1	3	2	1	5	6
SICN_009	CSF	-	VNI	α	1	1	1	3	2	1	5	6
SICN_011	Blood	+	VNI	α	1	1	1	4	2	1	5	4
SICN_012	CSF	+	VNI	α	1	3	1	5	2	1	1	5
SICN_013	Blood	+	VNI	α	1	1	1	4	2	1	5	4
SICN_014	CSF	+	VNI	α	1	1	1	4	2	1	5	4
SICN_015	CSF	+	VNI	α	1	1	1	3	2	1	5	6
SICN_017	Blood	+	VNI	α	1	1	1	4	2	1	5	4
SICN_018	CSF	+	VNI	α	1	23	10	3	4	1	1	93
SICN_019	CSF	+	VNI	α	1	3	1	5	2	1	1	5
SICN_021	Blood	-	VNI	α	1	3	1	5	2	1	1	5
SICN_022	Blood	U	VNI	α	1	1	1	3	2	1	5	6
SICN_024	Blood	+	VNI	α	1	1	1	3	2	1	5	6
SICN_025	CSF	-	VNI	α	1	1	1	3	2	1	5	6
SICN_026	Blood	+	VNI	α	1	1	1	3	2	1	5	6
SICN_027	CSF	+	VNI	α	1	1	1	3	2	1	5	6
SICN_028	CSF	+	VNI	α	1	3	1	5	2	1	1	5
SICN_029	CSF	-	VNI	α	1	1	1	3	2	1	5	6
SICN_030	Blood	+	VNI	α	1	3	1	5	2	1	1	5
SICN_031	Blood	+	VNI	α	1	1	1	3	2	1	5	6
SICN_032	CSF	+	VNI	α	1	1	1	4	2	1	5	4
SICN_033	Blood	+	VNI	α	1	1	1	3	2	1	5	6
SICN_034	Bone marrow	-	VNI	α	1	1	1	3	2	1	5	6
SICN_035	CSF	+	VNI	α	1	1	1	4	2	1	5	4
SICN_036	CSF	+	VNI	α	1	1	1	4	2	1	5	4
SICN_038	CSF	+	VNI	α	1	1	1	4	2	1	5	4
SICN_039	CSF	-	VNI	α	1	1	1	4	2	1	5	4
SICN_040	CSF	+	VNI	α	1	1	1	4	2	1	5	4
SICN_041	CSF	+	VNI	α	1	1	10	3	4	1	1	32
SICN_060	Blood	U	VNI	α	1	1	1	4	2	1	5	4
SICN_061	CSF	U	VNI	α	1	1	1	3	2	1	5	6
SICN_062	CSF	U	VNI	α	1	1	1	4	2	1	5	4
SICN_063	CSF	U	VNI	α	1	1	1	3	2	1	5	6
SICN_064	Blood	U	VNI	α	1	1	1	4	2	1	5	4
SICN_065	CSF	U	VNI	α	1	1	1	4	2	1	5	4
SICN_066	Blood	U	VNI	α	1	1	1	4	2	1	5	4
SICN_067	CSF	U	VNI	α	1	1	1	4	2	1	5	4
SICN_068	CSF	U	VNI	α	1	1	1	4	2	1	5	4
SICN_069	CSF	+	VNI	α	1	1	1	4	2	1	5	4
SICN_070	CSF	U	VNI	α	1	3	1	5	2	1	1	5
SICN_071	CSF	U	VNI	α	1	1	1	3	2	1	5	6
SICN_072	Blood	U	VNI	α	1	1	1	4	2	1	5	4
SICN_073	Blood	U	VNI	α	1	1	1	3	2	1	5	6
SICN_074	Blood	U	VNI	α	1	3	1	5	2	1	1	5
SICN_075	CSF	U	VNI	α	1	1	1	3	2	1	5	6

U = Unknown, CSF = Cerebrospinal fluid, ST = sequence type

The medical records of 35 patients with cryptococcosis were available. The patients’ ages ranged from 24 to 82 years (mean, 40.2 years) and most were males (65.71%). Thirty-one patients (88.57%) were HIV positive (Table 1).

### MLST analysis

**Fig 1:**
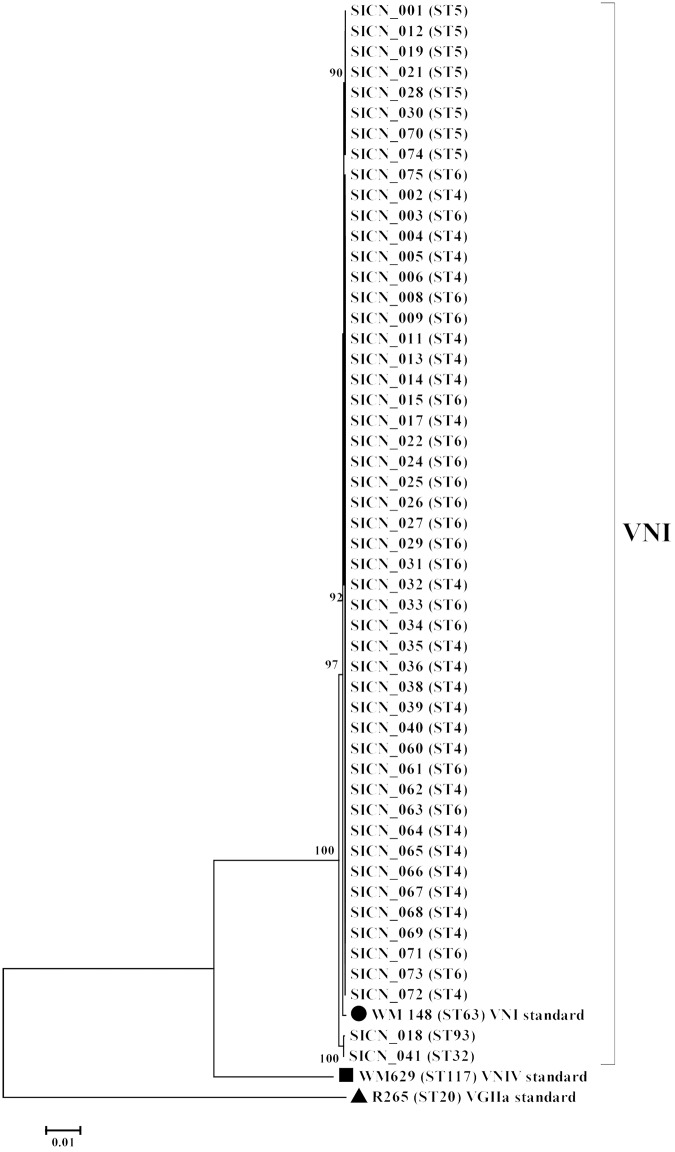
Phylogram of the *C*. *neoformans* isolates. Phylogram depicting the genetic relationships between the *C*. *neoformans* isolates based on neighbor joining analysis of the concatenated seven ISHAM consensus MLST loci using the program MEGA 6.06.

All isolates were identified as molecular type VNI (Table 2). The most common allele type of *CAP59*, *GPD1*, IGS1, *LAC1*, *PLB1*, *SOD1* and *URA5* gene was allele type 1 (100.00%), 1 (82.35%), 1 (96.08%), 4 (45.10%), 2 (96.08%), 1 (100.00%) and 5 (80.39%), respectively. The MLST analysis divided the 51 *C*. *neoformans* isolates into five STs: ST4 (45.10%, n = 23), ST6 (35.29%, n = 18), ST5 (15.69%, n = 8), ST93 (1.96%, n = 1), and ST32 (1.96%, n = 1) (Table 2). A phylogram of sequence types was shown in Fig 1.

### ST5 not associated with non-HIV patients in Thailand

Recent data showed a significant association between ST5 and non-HIV patients. Therefore, we expected a similar phenomenon among the Thai isolates. However, our results showed that ST5 was mainly detected in HIV-positive patients (5 out of 6 patients, 83.33%) unlike the previous reports [13, 14]. Moreover, the association of ST5 and HIV status was not evident in this study as there was no significant difference in the prevalence of HIV infection in ST5 and non-ST5 infected patients (P = 0.657) (Table 3).

**Table 3:** Association between sequence types, HIV status, and time.

Data	Sequence type			P-value
		ST5	Non-ST-5	
**HIV status**^#^	Positive (n = 31)	5 (16.13%)	26 (83.87%)	0.657
	Negative (n = 4)	1 (25.00%)	3 (75.00%)	
**Years**	2003–2005 (n = 231)	29 (12.55%)	201 (87.45%)	0.548
	2012–2014 (n = 51)	8 (15.70%)	43 (84.30%)	

^#^only data available in medical records were considered

### No change in ST distributions in Thailand in the last 10 years

After a national campaign of highly active antiretroviral therapy (HAART), the prevalence of cryptococcosis dropped significantly. According to our records, a 90% reduction in cases of cryptococcosis occurred in the past 10 years (data not shown). Therefore, we speculated that changes in the ST distributions may have occurred. However, our data showed no such change (P = 0.548), as shown in Table 3.

### High mortality was associated with tuberculosis and non-HIV strains

Because comorbidities may affect the efficacy of treatment for cryptococcosis, we investigated the effects of tuberculosis (TB) and HIV infection on treatment outcomes. As expected, patients with active TB had higher morbidity than those without TB (75% vs 25%, respectively; P = 0.031). However, surprisingly patients without HIV infection had higher morbidity than those with HIV (66.67% vs 33.33%, respectively; P = 0.041) (Table 4).

**Table 4:** Association between comorbidity (HIV or TB) and treatment outcome#.

Disease		Treatment outcome		P-value
		Cure	Death	
**TB**	Active (n = 4)	1 (25.00%)	3 (75.00%)	0.031
	Non active (n = 27)	18 (78.26%)	5 (21.74%)	
**HIV**	Positive (n = 22)	17 (77.27%)	5 (22.73%)	0.041
	Negative (n = 6)	2 (33.33%)	4 (66.67%)	

^#^only data available in medical records were considered.

Because comorbidities can complicate the investigation of cryptococcosis, we tested whether TB or HIV infections delayed the diagnosis of cryptococcosis. Our data showed that coinfection with TB or being HIV-negative delayed the diagnosis of cryptococcosis (1.9 and 2.3 times longer, respectively) (Table 5). The difference between the HIV-coinfected and uninfected patients was significant (P = 0.024). However, no significant difference between the TB-coinfected and uninfected patients was observed (P = 0.401).

**Table 5:** Association between comorbidity (HIV or TB) and time from symptom onset to disease diagnosis^#^.

Disease	Mean of time from symptom onset to disease diagnosis (days)		P-value
	Active/positive status	Non-active/negative status	
**TB**	42.50 (n = 4)	22.35 (n = 31)	0.401
**HIV**	18.74 (n = 27)	44.63 (n = 8)	0.024

^#^only data available in medical records were considered.

## Discussion

Cryptococcosis is usually considered an opportunistic infection, and is associated with acquired immune deficiency syndrome (AIDS) in 90% of the patients. A patient typically becomes infected by inhaling the spores of *Cryptococcus*, which primarily cause lung disease. The spores can then disseminate to other organs via the bloodstream, especially to the CNS. In this study, we found that the commonest source of cryptococcal isolates was the cerebrospinal fluid (56.86%), whereas 41.18% of the isolates were from blood and 1.96% from bone marrow, which is known to be involved in the pathogenesis of this organism. Our results are consistent with a previous study, in which more than 80% of specimens were isolated from cerebrospinal fluid [4].

*Cryptococcus neoformans* VNI is the molecular type most commonly found in Thailand and around the world [4, 7], and usually infects immunocompromised individuals.

Surprisingly, in 2008, it was reported that the majority (91.5%) of *C*. *neoformans* VNIc/ST5 isolates detected in China were from immunocompetent patients [15]. This phenomenon has also been reported in other areas of Far East Asia [4, 8, 16, 17]. Therefore, we undertook an epidemiological study of the putatively non-HIV-associated genotype, ST5, in Thailand, where the population is closely genetically related to that in Far East Asia. Our results are consistent with those of all previous studies throughout the world, in that more than 90% of the isolates were VNI [4, 18]. Our MLST analysis showed high prevalence of the ST4 and ST6 genotypes which is consistent with a previous study in Thailand [4]. A minor genotype, ST93 is the dominant sequence type in India and Indonesia [8]. A recent report showed that the other less common genotype, ST32, is also rarely (3.2%) found in Japan. [12]. These data demonstrate clear genetic variations among different Asian regions [4, 8, 16, 17]. A phylogram, constructed with a neighbor-joining analysis based on concatenated data from seven loci, showed that the five STs (ST 4, 5, 6, 32, and 93) detected in this study were genetically homogeneous. This clonality of *C*. *neoformans* was also confirmed by the fact that all the strains belonged to MATα.

Most strains were isolated from HIV-positive patients (88.6%), which is consistent with a previous study [4]. The male predominance (65.71%) is consistent with previous data collected in Thailand, which showed that more than 60% of cryptococcosis patients were male [4]. A slight increase in the mean age, from 30.5 years in 1997 to 32.4 years in 2004, has been reported [19]. A later study in 2013 reported a further increase in the average age to 37.97 years [4]. This trend was evident again in our study, where the average age had increased to 40.2 years. One explanation might be that HARRT improves the quality of patients’ lives and therefore increases their life spans [20].

Unlike cryptococcosis in Japan [16], China [13], and Korea [14], no association was evident between ST5 and non-HIV status in patients at Siriraj Hospital in this study. Only 15.69% of the clinical isolates were ST5 and 12.50% of the ST5 isolates were from an HIV-negative patient. It has been reported that ST5 is found in both HIV-positive and negative patients [8]. Our data confirms that the association between ST5 and non-HIV status is unique to Far East Asia. Although this association has been tentatively attributed to the specific genetic susceptibility of the Far East Asian population [8], our data suggest otherwise. Because the genetic backgrounds of the Fast East Asian and South East Asian populations are reportedly close [21], the specific genetic susceptibility theory is inconsistent with this difference in the ST5–HIV relationship in these two Asian populations. However, because the number of samples analyzed in the present study was limited, a larger study is required.

Coinfection with *Cryptococcus* and *Mycobacterium tuberculosis* is relatively difficult to treat. Our results confirm that comorbidity with tuberculosis reduces the treatment efficacy for cryptococcosis. There is evidence that *M*. *tuberculosis* and *Cryptococcus* synergistically suppress the immune system, and consequently reduce the signs and symptoms of patients, delaying their diagnosis and treatment [22]. Treatment also failed more frequently in HIV-negative patients in this study. A similar suggestion was proposed in another report, in which a delay in the diagnosis of cryptococcosis in non-HIV patients was attributed to the negative or low antigen titers in their samples [23]. These proposals are also supported by our finding that coinfection with TB or being a no HIV infection delayed the diagnosis of cryptococcosis. Therefore, the timely diagnosis of the disease, especially in TB endemic areas, is essential [24, 25].

In conclusion, the non-HIV-specific ST5 is still less common than other STs in Thailand. A phylogenetic analysis showed that the five STs detected were highly homogeneous. However, the relationship between the sequence type and the host’s HIV status is still unclear. The collection of more clinical strains is required to clarify this relationship.
